# Archaeal Protein Biogenesis: Posttranslational Modification and Degradation

**DOI:** 10.1155/2010/643046

**Published:** 2010-09-30

**Authors:** Jerry Eichler, Julie Maupin-Furlow, Joerg Soppa

**Affiliations:** ^1^Department of Life Sciences, Ben-Gurion University of the Negev, Beersheva, Israel; ^2^Department of Microbiology and Cell Science, University of Florida, Gainesville, FL 32611, USA; ^3^Institute for Molecular Biosciences, Frankfurt University, 60325 Frankfurt, Germany

The study of Archaea at the DNA and RNA levels has provided considerable insight into replication, transcription, and other information-associated events which are either unique to this remarkable group of organisms or which were later found to also occur in Bacteria and/or Eukarya. In contrast, largely due to a lack of suitable model systems and a limited number of appropriate molecular tools, considerably less was known about archaeal proteins in terms of their biogenesis, modification, trafficking, or degradation. In recent years, however, we have witnessed major advances in proteomics, successful in vitro reconstitutions, and the development of reporter systems compatible with extreme conditions. Relying on such tools, insight into different stages in the life of archaeal proteins has begun to accumulate. In this special issue of Archaea, we present a series of articles addressing the current state of understanding of selected facets of archaeal protein biogenesis and processing. 

An article by De Koning et al. discussing how fidelity in archaeal information processing at the DNA, RNA, and protein levels is achieved begins this special issue. Rother and Krzycki then address proteins containing the unusual animo acids, selenocysteine, and pyrrolysine, as related to the unique energy metabolism of methanogenic archaea. Soppa compares one specific posttranslational modification, acetylation, across evolutionary lines, while Botting et al. discuss the importance of lysine methylation in hyperthermophilic crenarchaeota. Questions related to protein assembly are considered when Iwasaki addresses the iron-sulfur world in hyperthermoacidophilic archaea.

Protein degradation is the focus of two articles in this special issue. Makarova and Koonin rely on comparative genomic analysis to reveal functional versatility of archaeal ubiquitin-like proteins, while Humbard et al. report on the phosphorylation and methylation of *Haloferax volcanii* proteasomal proteins.

Archaeal cell surface proteins undergo a variety of post-translational modifications. However, such proteins must first be targeted to and traverse the plasma membrane. Accordingly, Zwieb and Bhuiyan discuss the latest findings on the archaeal signal recognition particle targeting system. Storf et al. address questions related to the biogenesis of one class of membrane proteins, namely, lipoproteins. An article by Ellen et al. considers archaeal protein export and describes different cell surface structures, while a report by Jarrell et al. focuses on the S-layer glycoprotein and flagella as reporters of choice of different protein processing events. With this in mind, Peyfoon and colleagues describe the N-linked glycan decorating the S-layer glycoprotein of *Sulfolobus acidocaldarius*. Finally, Kaminski and Eichler consider the workings of AglD, one of the enzymes which is involved in *Haloferax volcanii* S-layer glycoprotein N-glycosylation.

In this, the first special issue of Archaea dedicated to archaeal protein biogenesis, we have tried to give the reader a sampling of the current research scene. It is our sincere hope that the community will find interest in the articles included here. More importantly, it is our intention that the work presented will stimulate other laboratories to begin studying questions related to archaeal protein biogenesis, hopefully in time for the next installment of this special issue. 

*Jerry Eichler* *Julie Maupin-Furlow* *Joerg Soppa*

